# Bartholin's abscess arising within hidradenoma papilliferum of the vulva: a case report

**DOI:** 10.1186/1757-1626-1-282

**Published:** 2008-10-29

**Authors:** Salvatore Docimo, Wonwoo Shon, David E Elkowitz

**Affiliations:** 1 Academic Medicine Fellow, New York College of Osteopathic Medicine, Old Westbury, NY, USA; 2 Department of Pathology, New York College of Osteopathic Medicine, Old Westbury, NY, USA

## Abstract

**Background:**

Hidradenoma papilliferum is an uncommon, benign, cystic, papillary tumor that occurs almost exclusively in the female anogenital region. Bartholin's abscess is also an anogenital cystic lesion caused by obstruction of Bartholin's duct with an overlying infection. Concomitant presentation of Bartholin's abscess and Hidradenoma papilliferum is unique.

**Case presentation:**

A 43-year-old African American woman presented with a painful cystic mass on the left labia majora. A preoperative diagnosis of Bartholin's abscess was made. During excision and draining, an additional tan-brown dermal nodule was removed which demonstrated histological features of Hidradenoma papilliferum.

**Conclusion:**

We present what we believe to be the first case of Bartholin's abscess arising in hidradenoma papilliferum and its clinical significance.

## Background

Hidradenoma papilliferum (HP) is an uncommon, benign, cystic, papillary tumor that occurs primarily in the vulva of Caucasian women, thought to arise from anogenital glands, and exhibits both eccrine and apocrine differentiation, with the latter being more common [1]. Bartholin's abscess is also an anogenital cystic lesion caused by partial or complete ductal obstruction with abscess formation subsequently following infection [2]. Concomitant presentation of Bartholin's abscess and Hidradenoma papilliferum is unique. We report to the best of our knowledge the first case of Bartholin's abscess arising in hidradenoma papilliferum.

## Case presentation

**Figure 1:**
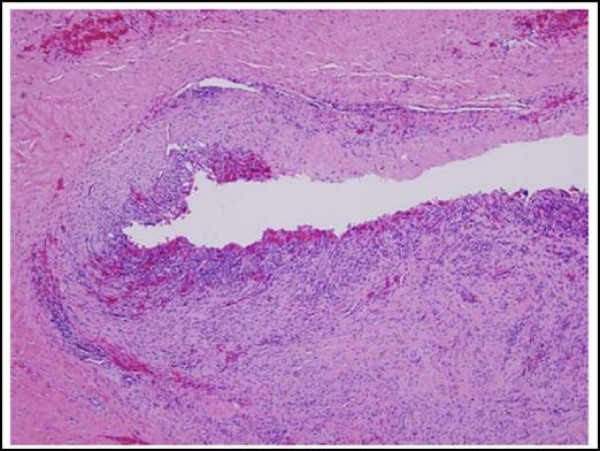
**Bartholin's abscess wall demonstrating fibro-necrotic tissues with acute and chronic inflammation.** (Hematoxylin-eosin stain; original magnification: ×10).

**Figure 2:**
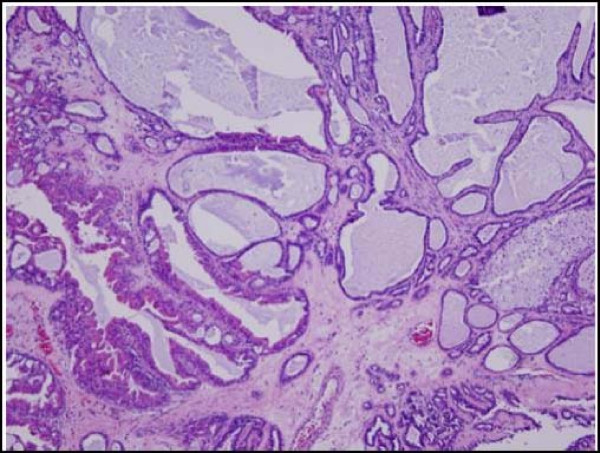
**Papillary neoplasm with cystic dilation.** (Hematoxylin-eosin stain; original magnification: ×10).

**Figure 3:**
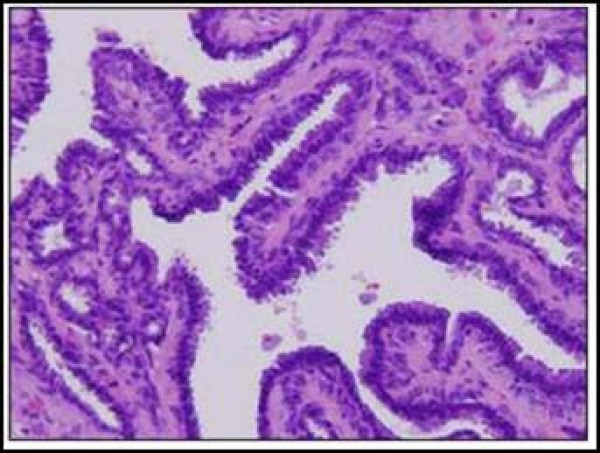
**The papillary projections and cystic areas are lined by basophilic cuboidal to columnar cells with outer compressed myoepithelial cells.** (Hematoxylin-eosin stain; original magnification: ×10).

**Figure 4:**
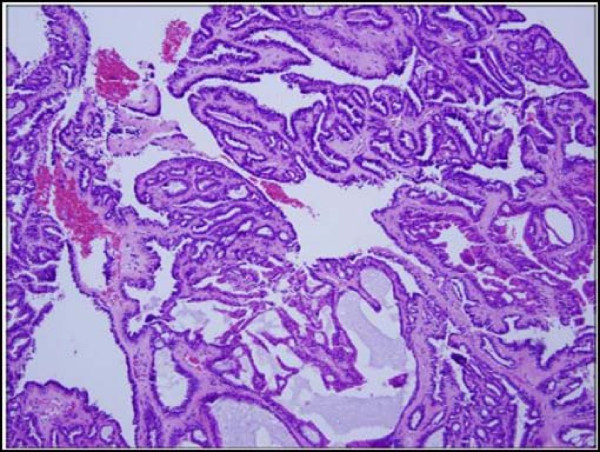
**Hidradenoma papilliferum.** Papillary neoplasm with foci of apocrine differentiation and cystic dilation. (Hematoxylin-eosin stain; original magnification: ×10).

A 43-year-old African American woman presented with a painful cystic mass on the left labia majora. Preoperatively, the lesion was diagnosed as Bartholin's abscess. During the excision and drainage, an additional 2.0 × 0.8 × 0.8 cm tan-brown dermal nodule was identified and submitted for histological analysis. Microscopic examination revealed multiple pieces of fibro-necrotic tissues (Fig. 1) associated with a well-circumscribed papillary neoplasm with cystic dilation (Fig. 2). The papillary projections and cystic areas were lined by basophilic cuboidal to columnar cells with outer compressed myoepithelial cells (Fig. 3). There were foci of active decapitation secretion and apocrine differentiation (Fig. 4). The diagnosis of Bartholin's abscess arising in hidradenoma papilliferum was made.

## Discussion

Previous literature has described instances of pathology occurring concurrently with Bartholin's abscess or cyst. Literature describes a case of cellular angiofibroma for which the preoperative diagnosis was Bartholin's glandular cyst [3]. Another case report described a vulvar mass that was preoperatively diagnosed as a Bartholin's duct cyst but found to be leiomyosarcoma, a rare gynecologic malignancy [4]. Although significant clinical correlations between hidradenoma papilliferum and Bartholin's abscess are not well understood at this point, the intimate association between these two lesions seen on histological examination suggests partial or complete obstruction of ductal drainage by the tumor mass. Thus, indicating Bartholin's abscess arising due to the presence of hidradenoma papilliferum.

Bartholin's glands are bilaterally located at the base of the labia minora and drain through 2- to 2.5-cm-long ducts that empty into the vestibule at the 4 o'clock and 8 o'clock positions [5,6]. Hidradenoma papilliferum usually presents as a slow-growing, small (2 mm to 3 cm) nodule which most commonly arise from the apocrine sweat glands between the labia majora and labia minora [7]. Woodworth et al. reported 38% of hidradenoma papilliferum originate from the labia majora and 26% originate from the labia minora [8]. Occurrence of hidradenoma papilliferum in the labia minora certainly presents the possibility of disrupting or blocking the ducts of Bartholin's glands which are located within the vicinity.

Due to hidradenoma papilliferum's mixed eccrine and apocrine features, cyst or abscess formation secondary to the disruption of ductal drainage should be considered [8]. Hidradenoma papilliferum should be present in the differential in the context of recurrent cysts/abscesses in the anogenital region [9]. The clinical differential diagnosis of a Bartholin's abscess, which includes mucous cysts, epidermal inclusion cysts, and hidradenoma papilliferum [9], demonstrates the importance of maintaining an awareness of differentiation between Bartholin's abscess and hidradenoma papilliferum.

Diagnostically, hidradenoma papilliferum tends to occur exclusively in post-pubescent white women between the ages of 30 and 70, occurring most commonly in the fourth decade [8,9]. Interestingly, our diagnosis of Bartholin's abscess arising in hidradenoma papilliferum was made in an African American woman.

Though it has been suggested surgical excision of Bartholin's abscess is unnecessary due to the low risk of Bartholin's gland cancer [10], our finding of hidradenoma papilliferum and other mentioned neoplasms demonstrates the need for an increased level of suspicion with a preoperative diagnosis of Bartholin's abscess. Gynecologic oncology referral is also supported in patients older than 40 years of age with a Bartholin's abscess to rule out adenocarcinoma [11].

While hidradenoma papilliferum is a benign tumor, malignant transformations to adenocarcinoma or adenosquamous carcinoma have been reported, although rare [12-16]. Therefore, a diagnosis of Bartholin's abscess without further investigation into the presence of hidradenoma papilliferum may put the patient as risk for a malignant process.

## Conclusion

We concluded the diagnosed Bartholin's abscess was most likely caused by obstruction of the duct of Bartholin's gland by hidradenoma papilliferum. A diagnosis of Bartholin's abscess should not signal a cessation in further investigation for other pathologies. The clinical presentation of hidradenoma papilliferum itself becomes increasingly indistinct when simultaneously occurring with other cystic lesions of the vulva. As noted previously, though rare, hidradenoma papilliferum does have a potential for malignant conversion. Thus, one should remain diligent in finding a cause of a Bartholin's abscess.

## Consent

Written informed consent could not be obtained. The patient was brought into the emergency room and treated as needed. However, consent was not obtained as the patient left the hospital quickly following treatment. The patient was lost to follow-up as she cannot be contacted due to her unique legal situation. Furthermore, no family contact information was made available. We, the authors, do not believe the patient would object to the writing of our report or the information provided. Lastly, the patient's confidentiality has been protected as no identifiers are given in the report except for age and gender.

## Competing interests

The authors declare that they have no competing interests.

## Authors' contributions

DE was involved in the intellectual analysis of the data and contributed to the writing of the manuscript. WS was involved in the intellectual analysis, carrying out of experimental work on the project, and editing. SD was involved in the intellectual analysis and the major contributor to the writing of this case report. All authors read and approved the final manuscript.

## References

[B1] Meeker JH, Neubecker RD, Helwig EB (1962). Hidradenoma papilliferum. Am J Clin Pathol.

[B2] Marzano DA, Haefner HK (2004). The Bartholin gland cyst: past present and future. J Lower Genital Tract Dis.

[B3] Silva AC, Nascimento AG, Da Silva CS, Murta EF, Adad SJ (2005). Cellular angiofibroma of the vulva: case report with clinicopathological and immunohistochemistry study. Sao Paulo Med J.

[B4] Dewdney S, Kennedy CM, Galask RP (2005). Leiomyosarcoma of the vulva: a case report. J Reprod Med.

[B5] Govan AD, Hodge C, Callander R (1985). Gynaecology illustrated.

[B6] Azzan BB (1978). Bartholin's cyst and abscess. A review of treatment of 53 cases. Br J Clin Pract.

[B7] Kaufman RH (1994). Benign diseases of the vulva and vagina.

[B8] Katz VL, editor (2007). Comprehensive Gynecology.

[B9] Crum CP, editor (2006). Diagnostic Gynecologic and Obstetric Pathology.

[B10] Visco AG, Del Priore G (1996). Postmenopausal Bartholin gland enlargement: a hospital-based cancer risk assessment. Obstet Gynecol.

[B11] Omole F, Simmons B, Hacker Y (2003). Management of bartholin's duct cyst and bland abscess. Am Fam Physician.

[B12] Veraldi S, Schianchi-Veraldi R, Marini D (1990). Hidradenoma papilliferum of the vulva: report of a case characterized by unusual clinical behavior. J Dermatol Surg Oncol.

[B13] Hashimoto K, Lever WF, Freedberg IM, Eisen AZ, Wolff K, et al (1999). Tumors of skin appendages. Fitzpatrick's Dermatology in General Medicine.

[B14] Elder D, Elenitsas R, Ragsdale BD, Elder D, Elenitsas R, Jaworsky C, et al (1997). Tumors of the epidermal appendages. Lever's Histopathology of the Skin.

[B15] Goette DK (1988). Hidradenoma papilliferum. J Am Acad Dermatol.

[B16] Stefanato CM, Finn R, Bhawan J (2000). Extramammary Paget disease with underlying hidradenoma papilliferum: guilt by association?. Am J Dermatopathol.

